# Organizational structure, climate, and collaboration between juvenile justice and community mental health centers: implications for evidence-based practice implementation for adolescent substance use disorder treatment

**DOI:** 10.1186/s12913-020-05777-3

**Published:** 2020-10-08

**Authors:** A. Johnson-Kwochka, A. Dir, M. P. Salyers, M. C. Aalsma

**Affiliations:** 1 Psychology Department, Indiana University-Purdue University Indianapolis (IUPUI), Indianapolis, IN USA; 2 Faculty, Adolescent Behavioral Health Research Program, Indiana University School of Medicine, Indianapolis, IN USA; 3 Faculty, Department of Psychiatry, Indiana University School of Medicine, Indianapolis, IN USA; 4 Faculty, Psychology Department, Indiana University-Purdue University Indianapolis (IUPUI), Indianapolis, IN USA; 5 Faculty, Department of Pediatrics, Division of Adolescent Medicine, Indiana University School of Medicine, Indianapolis, IN USA

**Keywords:** Substance use disorders, Juvenile justice, Community mental health, Implementation barriers

## Abstract

**Background:**

Substance use disorders are prevalent among youth involved with the criminal justice system, however, evidence-based substance use disorder treatment is often unavailable to this population. The goal of this study was to identify barriers to effective implementation of evidence-based practices among juvenile justice and community mental health organizations through the lens of an adopter-based innovation model.

**Methods:**

In this mixed-methods study, qualitative interviews were conducted with *n* = 15 juvenile justice staff and *n* = 14 community mental health staff from two counties implementing substance use services for justice involved youth. In addition, *n* = 28 juvenile justice staff and *n* = 85 community mental health center staff also completed quantitative measures of organizational effectiveness including the implementation leadership scale (ILS), organizational readiness for change (ORIC), and the implementation climate scale (ICS).

**Results:**

Organizationally, staff from community mental health centers reported more “red tape” and formalized procedures around daily processes, while many juvenile justice staff reported a high degree of autonomy. Community mental health respondents also reported broad concern about their capacity for providing new interventions. Staff across the two different organizations expressed support for evidence-based practices, agreed with the importance of treating substance use disorders in this population, and were enthusiastic about implementing the interventions.

**Conclusions:**

While both community mental health and juvenile justice staff express commitment to implementing evidence-based practices, systems-level changes are needed to increase capacity for providing evidence-based services.

## Background

Substance use is prevalent among justice-involved youth; up to one-third meet criteria for a substance use disorder [1, 2] and juvenile offenders who experience substance use problems are more likely to remain involved with the justice system [3]. Ideally, both juvenile justice and community mental health organizations can work in concert to fully support the youth, reducing both substance use and the likelihood of recidivism [4]. More specifically, juvenile justice staff serve as gatekeepers, initially identifying youth in need of services and connecting them to appropriate services offered by community mental health systems. However, many justice-involved youth do not receive needed treatment; as few as 5% of eligible high-risk offenders receive evidence-based treatment annually in the United States [5]. One reason for the gap between those who need treatment and those who receive treatment is due to [1] juvenile justice organizations’ lack of appropriate substance use screening and [2] community mental health centers’ lack of available evidence-based substance use treatments [6]. Further complicating this is the difficulty in communication and collaboration between juvenile justice and community mental health centers.

**Fig. 1:**
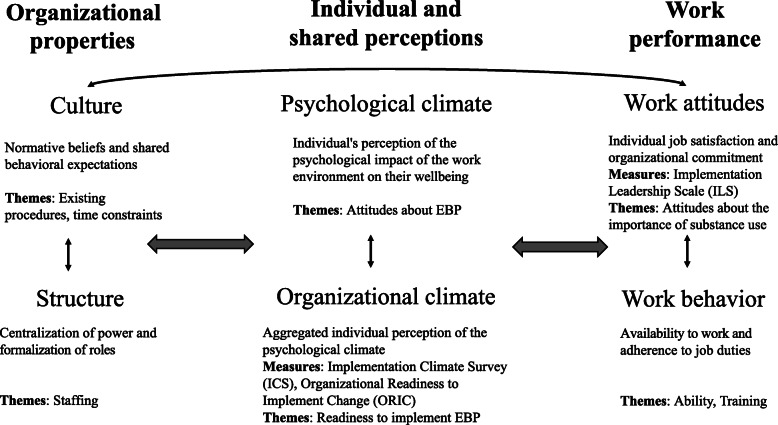
A model of organizational social context. Note: This figure has been adapted from Glisson, 2002. Examples of themes and measures are provided; themes shown here are not an exhaustive list of those described in this paper. An organizational social context is composed of organizational properties, individual and shared perceptions, and work performance. Organizational properties are composed of culture and structure. Individual and shared perceptions are composed of the psychological and organizational climates. Work performance is composed of work attitudes and work behavior

Community mental health centers and juvenile justice organizations are faced with unique challenges that impact the implementation and sustainment of EBPs. One useful way to consider how community mental health and juvenile justice organizations differ in their ability to implement EBPs is through the adopter-based theory of innovation diffusion [7, 8]. In this model, *organizational properties* impact staff members’ *individual and shared perceptions* of the organization and its work, which in turn impact employees’ *work performance*. *Organizational properties* are essentially the way things are done at each organization, and include the organizations’ culture (i.e., normative beliefs and shared behavioral expectations [9]) and structure (i.e., the centralization of power and staff/employee hierarchy). *Individual and shared perceptions* refer to employees’ own perceptions of the workplace (psychological climate) as well as the collective perceptions of the workplace (organizational climate)*;* for example, staff views about the organization’s readiness to implement an EBP, as well as organization-wide view about the importance of an EBP [10]. *Work performance* is composed of employee’s behavior (e.g., participating in trainings, successfully completing work) and attitudes about their work (e.g., attitudes about managers’ ability to lead EBP implementation). A model of these relationships (adapted from [7]) is shown in Fig. 1.

The adopter-based theory of innovation diffusion suggests that the adoption, implementation, and adaptation of a new and innovative technology (i.e., EBP) is primarily a function of the organization’s overall social context, which is a function of the organizations’ properties, individual and shared perceptions, and work performance. One aspect of social context that impacts adoption, implementation, and adaptation is an organization’s *culture*. Cultures that support achievement, self-actualization, humanistic behaviors, and affiliation with coworkers promote innovation and are more likely to adopt state of the art technologies [11]. In contrast, cultures whose norms are characterized by approval, dependence, avoidance, competition, power, and perfectionism, are more likely to reject or resist innovation [12]. New EBPs are inherently vulnerable to the organizational culture in which they are introduced, and are just as likely to be modified and re-invented to fit the organization’s context as they are likely to change the organizations where they are implemented.

Extant research on organizational social context in mental health services has found that children served by case management units with constructive organizational cultures (e.g., characterized by expectations that case managers would be mutually supportive, develop their individual abilities, maintain positive interpersonal relationships, and be motivated to succeed) were much more likely to receive needed mental health care [13]. Research also suggests that organizations with more constructive cultures have lower turnover rates among staff members, have more positive attitudes towards EBPs, and are able to sustain new treatment programs longer than organizations with more defensive cultures [14–17].

In this paper, we examine differences in organizational social contexts between community mental health centers and juvenile justice organizations in two rural midwestern counties in the early stages of implementing universal substance use screening and evidence-based interventions designed to reduce substance use and criminal recidivism among youth in the juvenile justice system. Our aim is to identify barriers and facilitators to [1] implementation of evidence-based substance use practices in juvenile justice and community mental health organizations and [2] collaboration across organizations by examining these processes through the lens of the adopter-based innovation model.

## Methods

The current analysis is a part of a broader implementation effort to identify justice involved youth with substance use treatment needs and engage them in evidence-based substance use treatment. Below, we provide brief context of the overall study and EBP implementation; more information can be found in the published study protocol [18]. In order to guide implementation efforts, we interviewed staff from all participating sites to gather perspectives on the value of EBP for substance use treatment and implementation of the current interventions; we report the methods of our qualitative analyses following the (COREQ) guidelines for reporting qualitative research [19]. We also collected survey data about organizational climate and work attitudes (see Fig. 1). Our university’s institutional review board approved the study. All participants gave informed consent for participation.

### Setting

The study took place at community mental health centers and juvenile justice organizations from rural and suburban counties in a Midwestern state; existing research shows that although a numerical minority of justice-involved youth are served in rural settings, these youth have unique needs [20, 21]. Prior research also suggests that rural areas may have fewer resources and, regardless of geographical setting, community mental health and juvenile justice organizations face similar challenges in collaborating [22]. County 1 had a metropolitan area with fewer than 250,000 people, while county 2 had a metropolitan area with fewer than 40,000 people, and was not adjacent to any larger metropolitan areas [23]. Seventy-five percent of the population in county 1 identified as white, non-Hispanic, while 88% of county 2 identified as white and non-Hispanic. In county 1, the community mental health center employed approximately 54 clinicians serving adolescents, while the juvenile justice organization employed 6 staff working directly with adolescents. In county 2, the community mental health center employed approximately 39 clinicians who work with adolescents while the juvenile justice organizations employed 20 staff who worked with adolescents.

### Evidence-based practices implemented

For the overall study, juvenile justice organizations implemented universal substance use screening for youth at intake, and the partner community mental health organizations implemented two substance use treatment interventions for participating justice-involved youth (described below), stratified by risk level [19]. We interviewed participants during the pre-implementation phase prior to each organization receiving training and consultation in the EBPs that they planned to implement; both organizations understood the basics of each intervention (e.g., substance use screening, motivational interviewing, cognitive-behavioral therapy), but were not familiar with the specifics.

#### Juvenile justice substance use screening

Juvenile probation officers were trained to screen all youth who completed the intake process using the CRAFFT [24], which consists of 6 yes or no questions about substance use. Research team members were notified when youth scored in the warning range on the CRAFFT and determined (based on the CRAFFT score) what level of care would most benefit the youth (i.e., Teen Intervene or ENCOMPASS). Parents and youth were then contacted to participate in the study.

#### Community mental health system substance use treatment

##### Teen intervene

For youth participants who scored in a low or moderate substance use risk level, community mental health organizations implemented Teen Intervene [25, 26], which consists of three to six sessions, delivered by bachelor’s level intervention specialists, who receive a one-time initial training and monthly meetings for ongoing training and consultation.

##### Encompass

For youth participants who scored in a high substance use risk level, partner community mental health organizations implemented ENCOMPASS [27–29], which consists of 16 sessions delivered weekly by master’s level therapists, who received in-person training and weekly consultation by phone (see 19 for further information regarding interventions implemented).

### Participants

Authors 1 and 4 conducted qualitative interviews with a subset of juvenile justice staff (*N* = 15, including probation officers, judges and intake staff) and community mental health center staff (*N* = 14, including case managers, therapists, and administrators). Author 1 had not previously met any of the research participants, while author 4 had collaborated with juvenile justice staff on previous projects related to criminal justice reform. Collaboration with the research team with both community mental health centers was newly established with both community mental health centers through this project. Administrators from both juvenile justice and community mental health centers agreed to have staff involved in the project because substance use treatment was a significant concern for both counties. An administrator from each organization provided a list of contact information for staff members who were familiar with or working on the implementation project, and a graduate research assistant contacted a randomly selected subset of these staff members to see if they were interested in participating and to set up an interview at their convenience. Our goal was to interview at least 5 staff members at each site, including both managers and non-managers; this was a convenience sample, with the goal of attaining data saturation across topic areas and job roles. All staff members who were contacted agreed to participate in interviews. Juvenile justice center staff were overrepresented as most staff members were directly involved with the project (e.g., overseeing probation officers or administering the CRAAFT at intake) while proportionally fewer community mental health center staff oversee or provide direct services to arrested youth. All interviews were voluntary and were audio-recorded. Because documentation of written informed consent would be the only identifiable connection to the research participant, interview participants provided verbal consent for participation, as was approved by the University’s IRB. Interviews lasted approximately 45 min and were conducted over the phone. Staff were not compensated for their time participating in interviews. Prior to conducting the interview, staff were told that researchers were primarily interested in learning about their current perspective on the availability and effectiveness of substance use disorder treatment for adolescents in their communities, as well as their thoughts about implementing EBPs as part of the current research study.

In addition to the qualitative interviews, we asked juvenile justice and community mental health center staff to complete brief surveys about their organization’s culture and attitudes toward EBP. In order to participate in the survey, staff had to work with adolescents or be in a leadership role. Participants included 28 juvenile justice staff (96.6% of those who were eligible) and 85 community mental health center staff (86.7% of those who were eligible). Surveys were administered via Qualtrics through an anonymous email link that research staff sent to eligible participants. Prior to completing the survey, prospective participants reviewed a study information sheet and provided anonymous informed consent by indicating that they would like to participate in the study, as approved by the University’s IRB. Surveys took less than 30 min to complete; staff were not compensated for participating in the survey.

### Measures

#### Qualitative interview guides

The research team developed the semi-structured interview guides to elicit participants’ perspective on substance use treatment and implementation of the interventions (See Additional file 1). Interview domains included familiarity with the interventions, availability of substance use treatment, existing referral processes between juvenile justice and community mental health, and participant perspectives on how to improve the working relationship between organizations. Members of the research team met to discuss the interviews early on in the data collection process and refine the interview guide to ensure quality data collection. In addition, members of the research team met throughout the data collection process to discuss emergent themes, discuss data saturation, and determine if additional interviews were needed. For the purpose of this study, we focused our analyses on themes regarding the organizational social context (see Fig. 1) of the juvenile justice and community mental health centers across both sites; we also provide examples pertaining to the larger study when they illustrate the social context of each organization. Participants were encouraged to share their own perspective, and the interviewer asked additional probing questions if necessary.

#### Survey measures

Surveys focused on participants’ views about organizational leadership in implementing new practices, organizational readiness to implement change (e.g., evidence-based practices), openness to EBP, and experience with the interventions using validated measures described below (see Fig. 1). None of these survey measures are under license.

#### Implementation leadership

Given the crucial role that organization leaders play in planning and supporting their staff in implementing new EBPs [30], implementation leadership assesses the degree to which a leader is capable of facilitating EBP implementation, and was measured using the Implementation Leadership Scale (ILS), a 12-item scale with four subscales measuring proactive leadership, knowledgeable leadership, supportive leadership, and perseverant leadership. Response options range from 1 to 5, with higher mean scores on each subscale denoting greater evidence of this leadership quality. Confirmatory factor analysis supports a 4-factor structure, and the ILS has demonstrated excellent internal consistency (α = .96 in our sample) as well as convergent and discriminant validity [31]. This scale was used to assess *work attitudes* in the model of organizational social context (see Fig. 1).

#### Implementation climate

Implementation climate was measured using the Implementation Climate Scale (ICS). The ICS is an 18-item measure of an organization’s strategic climate for the implementation of EBPs; the scale asks that organization supervisors rate their own behavior, and staff member rate their supervisors’ behavior. It has six subscales: focus on EBP, educational support for EBP, recognition for EBP, rewards for EBP, selection for EBP (i.e., selecting staff who value and have had experience with EBPs), and selection for openness (i.e., selecting staff who are open and can adapt to change), with response options from 1 to 5 and higher mean scores denoting greater leadership support for EBPs. Confirmatory factory analysis supports the 6-factor structure, and additional analyses support the reliability (α = .93 in our sample) and construct validity of the ICS [32]. This scale was used to assess individual and shared perceptions of the *organizational climate*.

#### Organizational readiness to change

Organizational readiness to change was measured using the Organizational Readiness to Implement Change scale (ORIC), a 12-item scale. The ORIC scale was developed based on Weiner’s theory of organizational readiness for change; consistent with this theory, confirmatory factor analysis supports two correlated factors (change commitment and change efficacy), and additional analyses support high internal consistency ( [33]; α = .97 in current sample). Response options range from 1 to 5, with higher mean scores indicating greater agreement that the organization is ready to implement the EBP. This scale was also used to assess individual and shared perceptions of the *organizational climate*.

### Data analysis

For the purposes of the current paper, we gathered quantitative and qualitative data to measure culture, psychological and organizational climate, work attitudes, and work behavior among juvenile justice and community mental health organizations. We analyzed qualitative and quantitative data simultaneously, and triangulated data across methodologies to assess for data convergence [34, 35].

For the quantitative data, we calculated descriptive statistics for each measure, and used t-tests to compare means between community mental health and juvenile justice organizations. Qualitative interview audio files were uploaded to Rev.com for transcription. Transcripts were then de-identified and uploaded to NVivo, a qualitative analytic software program, for coding and analysis [36]. Qualitative codes were developed by the research team using a combination of a priori categories based on the research questions and interview guides, as well as themes that emerged through inductive review of the interview transcripts [37]. Transcripts were coded in a multistage process. Initial coding by the graduate research assistant indexed transcripts according to the themes in the interview guide. Examples of initial codes included communication challenges, importance of substance use treatment, and excitement about interventions. Members of the research team met regularly to identify, discuss, and refine emerging themes in participants’ perspectives on implementation and working with other organizations (i.e., juvenile justice or community mental health). We identified a full set of codes by examining this data through the lens of diffusion of innovations [8]; see Table 1 for examples of codes and their corresponding quotations. Using this full set of codes, we conducted focused coding on all the transcripts and compared juvenile justice and community mental health responses to understand organizational differences [38]. Finally, we categorized information from survey scales and subscales according to the organizational level (i.e., organizational properties, individual and shared perceptions, work performance) that they informed and integrated quantitative and qualitative data using concurrent triangulation mixed methods analysis to form a more comprehensive picture of each level [39].

**Table 1:** Demographics by data and organization type

**Interview Respondents**	**CMHC** **(*N*** **= 14)**	**JJ** **(*N*** **= 15)**	**All** **(*N*** **= 29)**
Gender (N, % female)	10 (71.4%)	9 (60.0%)	19 (65.5%)
Race (N, % white)	13 (92.8%)	14 (93.3%)	27 (93.1%)
Ethnicity (N, % Non-Hispanic/Latino)	14 (100%)	14 (93.3%)	28 (96.6%)
Age (N, % between 26 and 35)	NA	6 (40.0%)	10 (34.4%)
Length of time in current position (N, % less than 1 year)	NA	NA	7 (24.1%)
Length of time at current agency (N, % less than 1 year)	NA	NA	NA
Education (N, % with at least bachelor’s degree)	14 (100%)	15 (100%)	29 (100%)
Job satisfaction (N, % at least satisfied)	14 (100%)	15 (100%)	29 (100%)
**Survey Respondents**	**CMHC** **(*N*** **= 85)**	**JJ** **(*N*** **= 28)**	**All** **(*N*** **= 113)**
Gender (N, % female)	68 (80.0%)	25 (89.3%)	93 (82.3%)
Race (N, % white)	72 (84.7%)	26 (92.8%)	98 (86.7%)
Ethnicity (N, % Non-Hispanic/Latino)	82 (96.5%)	27 (96.4%)	109 (96.4%)
Age (N, % between 26 and 35)	35 (41.2%)	8 (28.6%)	43 (38.1%)
Length of time in current position (N, % less than 1 year)	26 (30.6%)	7 (25%)	33 (29.2%)
Length of time at current agency (N, % less than 1 year)	21 (24.7%)	NA	25 (22.1%)
Education (N, % with at least bachelor’s degree)	67 (78.8%)	19 (67.9%)	86 (76.1%)
Job satisfaction (N, % at least satisfied)	71 (83.5%)	27 (96.4%)	98 (86.7%)

Note: NA indicates data not available due to cell size below 5 participants

## Results

Demographic information on respondents from both the qualitative interviews and online surveys indicated that participants were primarily female and identified themselves as white or Caucasian (See Table 1). Most participants (and all interview participants) had at least a bachelor’s degree and reported high job satisfaction. There were few differences in demographics between interview and survey respondents, although interview respondents reported a longer tenure at their organizations, likely because we purposefully interviewed participants in leadership positions at each organization. Additionally, there were few differences in demographics between juvenile justice and community mental health organizations, although the latter appeared to experience higher turnover (i.e., had fewer participants with job tenure of more than a year). Below, we describe emergent themes in the two organizational systems from qualitative interview data and corresponding quantitative data from surveys organized according to the diffusion of innovations model (See Table 2) which speak to differences between the two organizations. Descriptive statistics for all quantitative measures (broken down by organization) are available in Table 3. We also discuss specific themes around barriers to collaboration between the two organizations that emerged in the interviews.

**Table 2:** Data organization within the Diffusion of Innovations Model

Codes and Measures	Example Quotations	
*Organizational Properties*	*Community Mental Health*	*Juvenile Justice*
Staffing	“Staffing is an issue. If you were to say to me, ‘tomorrow you’re going to have a consistent flow of referrals, are you able to meet the need?’ I would say probably not. We’re working really hard on that.” – Administrator, County 2	“We can’t get a case manager for the kids. If we do get them plugged into therapy, it seems like often the therapists leaves and they have to start all over again.” – Administrator, County 1
Time Constraints	“My days are pretty full. So when am I going to schedule this conference call [for EBP supervision]? The program itself, I don’t really have any concerns about.” – Therapist, County 1	“I don’t have any concerns about [adding substance use screening to intake appointments], other than the fact that, because right now we schedule an intake appointment, and so we schedule those where we’ve got time to do those, and so as the intake process moves along and police start bringing kids straight from the arrest, adding more time to the intake process is a little concerning.” – Probation Officer, County 1
Existing Procedures	“Typically we will get a referral through their probations electronic system asking us to complete an intake, which includes a treatment plan and a psycho-social evaluation. A diagnosis. A mental status exam. A violence risk screen.” – Therapist, County 1	“We’ve never done a formal assessment of when [substance use] is experimentation versus casual use versus more problematic use, so that has always been a struggle, and quite frankly, because we don’t use a systematic assessment, it has always been left to personal impression.” – Probation Officer, County 2
*Individual and Shared Perceptions*	Community Mental Health Centers	Juvenile Justice
Implementation Climate Scale (ICS)	Assesses staff perceptions of an organization’s focus on and support for evidence-based practices. See Table 3 for scale results across organizations.	
Organizational Readiness to Implement Change (ORIC)	Assesses staff perceptions of an organizations’ readiness to implement a new evidence-based practice. See Table 3 for scale results across organizations.	
Attitudes about importance of substance use	“I think [addressing adolescent substance use] it’s very important. I do think it’s something that has become a lot more rampant. It seems they slip through the crack sometimes, especially with adolescents at like 12, 13, 14. Most of the time, I was seeing that they were already smoking pot.” – Case manager, County 1	“[Treating substance use among youth] is very important, because if we don’t treat it now, they’re just going to keep continuing using and that just causes more problems for them and their families.” – Probation Officer, County 2
Attitudes about EBP	“I think [the brief interventions] will definitely be beneficial. It is good for us to see how any model that’s created, to see if it actually works. If we’re following the model, is there success from it? I think that’s important.” – Therapist, County 1	“I am very excited about getting kids the help that they need and identifying the right kids who are willing and ready to accept that help.” – Administrator, County 1
*Work Performance*	*Community Mental Health*	*Juvenile Justice*
Ability	“[This site] is big and we are used to a variety of things going on all at once. And so, adding this won’t cause a major disruption. And we’ve implemented evidence-based practices before.” – Administrator, County 1	“We’ve been doing [substance use screening] here for a while now. So, we’ve all gotten pretty comfortable with it. I feel like having a more streamlined version like what [the researchers] have can be beneficial for sure because it’s very straightforward.” – Probation officer, County 1
Training	“We had a discussion this morning about setting up training for case managers to feel more confident and develop some competency about dealing with [substance abuse].” – Administrator, County 2	“if we don’t have a [substance use oriented] group starting, the recovery coach piece is all we’ve got [to refer youth to], or to meet individually, just with an individual therapist, who may or may not have any substance abuse specific training” – Probation officer, county 2
Implementation Leadership Scale (ILS)	Assesses staff perceptions of a leader’s ability to implement a new evidence-based practice. See Table 3 for scale results across organizations.	
*Collaboration*	*Community Mental Health*	*Juvenile Justice*
Communication	“I have found that [the probation officers] are pretty open with communication. The process typically works that we get a referral. If we have a group getting ready to start, we get the kids into the group, and if not, we just work on recovery coaching until the next group starts. ”– Case manager, County 1	“Case managers are always willing and open to talk and chat and try to be involved. As far as from a therapist standpoint, we don’t get a whole lot of support if it’s not during business hours.” – Administrator, County 1
Collaboration	“I do have relationships with probation and the local judges. I’ve worked in this field for about 10 years and have gone in front of the judges on multiple occasions and they’re all real people, understanding and empathetic to our clients and really strive to ensure that children have the best opportunity.” – Therapist, County 2	“I appreciate what they do, what they are trying to do for our community. I admire the work that they do, and I think they do a pretty good job of putting the right people in the right places, from what I know.” – Administrator, County 2

Note: Quotes have been edited for clarity and readability. “Administrator” denotes anyone in a leadership role at an organization

**Table 3:** Organizational and implementation measures by organization type

	Juvenile Justice		Community Mental Health				
	*M*	*SD*	*M*	*SD*	***t***	***df***	***P***
**Implementation Climate Scale (ICS)**^**1**^							
Focus on Evidence-Based Practice	4.5	0.7	4.1	0.8	−1.9	100	.06
Educational Support for Evidence-Based Practice	4.3	0.8	3.8	0.9	−2.2	99	.03*
Recognition for Evidence-Based Practice	3.7	1.3	3.8	1.0	.26	93	.79
Rewards for Evidence-Based Practice	2.0	1.4	3.2	1.3	3.6	94	.00*
Selection for Openness	3.9	0.9	3.9	0.9	−.07	99	.94
**Implementation Leadership Scale (ILS)**^**2**^							
Proactive	3.9	1.1	3.5	1.1	−1.9	95	.07
Knowledgeable	4.1	1.1	3.9	0.9	−.81	95	.42
Supportive	4.2	0.9	4.2	0.9	.00	96	.98
Perseverant	4.1	0.9	4.0	0.8	−.17	95	.86
**Organizational Readiness to Implement Change (ORIC)**^**3**^	4.1	0.8	4.1	0.8	−.56	100	.58

^1^Higher scores indicate higher frequency. (min = 1, “not at all,” max = 5, “very great extent”)

^2^Higher scores indicate greater evidence of this trait (min = 1, “not at all,” max = 5, “very great extent”). Note that supervisors are rating themselves, and staff members are rating their supervisors

^3^Higher scores indicate greater agreement that the organization is ready to implement the EBP (min = 1, “disagree,” max = 5, “agree”)

Note: We excluded one subscale from the ICS, “selection for evidence-based practice” due to missing data from community mental health participants

### Organizational properties

Organizational properties include culture (i.e., the normative beliefs and shared behavioral expectations in an organization unit) and structure (i.e., the centralization of power and formalization of roles in an organization). Relating to the structure of each organization, community mental health case managers and therapists had formalized procedures for many of their daily processes, and reported little independence or autonomy outside of specific clinical decisions. Several clinicians spoke about the high amount of “red tape” (i.e., highly specific rules and procedures required for many of their daily tasks), and administrators reflected on the necessity of adhering to funding requirements from multiple state and federal agencies. One administrator from a juvenile justice site highlighted the difference in organizational structure, stating that “community health centers are like battle ships. The Juvenile justice system is like a speedboat, so it’s just a difference in how we move about and navigate care and treatment. The community mental health centers have a whole lot more red tape.” In contrast to community mental health center staff reporting specific procedures and timelines that guided their decision making, Juvenile justice staff from both sites reported a high level of autonomy and independence in making decisions about cases and procedures, including an ability to have the court order treatment for justice-involved youth. In terms of organizational culture, probation officers reported a high degree of flexibility, and had developed individual ways of doing things (e.g., ways of making referrals to the community mental health center). For example, one probation officer described tailoring the standard process for making referrals to her own preferences by completing “the written referral, and I will call out and say hey, [contact at community mental health center], I am sending you over a referral, can you go ahead and set them up for an appointment.” As further evidence for their sense of autonomy (and impacted by both the structure and culture of the organization), probation officers reported that they did not meet for regular, structured meetings with supervisors or co-workers often because they worked on different schedules or simply didn’t need to regularly meet to discuss cases. One probation officer reflected that theirs was “a 24-hour facility, so [the supervisor] doesn’t meet with us mainly because of scheduling.”

Another prominent theme among community mental health respondents was concern about having enough time to devote to clinical and administrative work, as well as being able to adequately bill for time spent on implementation and delivery of new interventions that require intense supervision and training; these concerns stem both from the organization’s culture, or shared behavioral expectations, and the formal structure that maintains the organization’s financial viability. As an example, one administrator reflected that “therapists are not easy to come by, especially in community mental health. So [the therapists] devoting an extra hour a week [to supervision calls for this project], every minute adds up. That might be a challenge.” Juvenile justice staff did not report concerns about understaffing at their organizations or about having enough time for implementing the intervention; it should be noted that due to the nature of the intervention (as described in the Methods), the burden of implementation was much lighter for Juvenile justice organizations than for community mental health centers.

### Individual and shared perceptions

This domain is made up of the psychological and organizational climate of each organization; the psychological climate captures individual staff members’ perceptions of the impact of the work environment on their wellbeing at work, while the organizational climate captures shared perceptions about organizational culture and values. Here, we focus primarily on the ways in which individual and shared perceptions about the work environment might effect EBP implementation. In terms of psychological climate, respondents across organizations perceived and expressed broad effectiveness at work. Among community mental health respondents, all expressed high focus on EBPs (community mental health ICS Focus on EBP mean = 4.1 out of possible 5, SD = 0.8; See Table 3), and respondents reflected their perception of the organizational climate, speaking of a sense that their organization was ready to implement the EBP (community mental health Organizational Readiness for Implementing Change (ORIC) mean = 4.1, SD = 0.8). For example, one administrator reflected that people working on this project from her organization were “enthusiastic” and “excited,” adding that “we are used to a variety of things going on all at once. And so, adding this won’t cause a major disruption. And we’ve implemented EBPs before.”

Similarly, juvenile justice respondents perceived that their organizations were ready to implement the screening practices (ORIC mean = 4.1), and that this would not be especially burdensome. Respondents from all organizations agreed that their organization was focused on providing evidence-based services (Juvenile justice ICS Focus on EBP mean = 4.5, SD = 0.7). On the ICS, juvenile justice and community mental health organizations differed only in that respondents from community mental health centers endorsed significantly greater Rewards for EBP, while respondents from juvenile justice organizations endorsed significantly higher Support for EBP. These differences may be explained by structural factors, i.e., that juvenile justice organizations needed to devote less time to implementing the practice, and that community mental health centers had more built-in structure and protocols that necessitated the use of EBP.

Reflecting the psychological climate, as well as themes also evident in the organizational properties, masters-level clinicians in particular expressed worries that they might not have enough time (e.g., to add an hour of supervision for the intervention to their week). In concert with this theme, clinicians and clinic administrators expressed excitement about utilizing case managers’ skills for clinical interventions. One case manager expressed that he and his colleagues “really [wanted] to do this” and that he felt like he could effectively deliver the brief interventions. Meanwhile, Justice staff viewed the screening tool as a way to better serve youth and their families and more accurately assess for substance use risk. One probation officer noted that his goal was to “screen everyone fairly and impartially,” adding that:

“We’re not going to be selective as to the number of referrals. We’re not going to be selective as to the offense. We’re going to treat all kids the same, and let the tools determine the next steps. I think it gives us the opportunity to not only look at risk but need across a few different tools, so if a kiddo maybe scores low in criminal risk factors but high need in other areas, it’s going to help sharpen our tools as far as the way that we refer and serve families.”

Another administrator added that “the more assessments that we can do, or tools that we have to make substance abuse referrals, I think the better off we are.”

### Work performance

Work performance consists of work attitudes and work behavior, and includes themes related to individual-level job satisfaction, organizational commitment, ability, and adherence to procedures. At this level, individual respondents from all organizations endorsed a strong attitudinal commitment to providing substance use services and also supported the idea of implementing this EBP for justice-involved youth who struggle with substance use. Similarly, all juvenile justice staff expressed work attitudes emphasizing the importance of treating and preventing substance use among justice-involved youth. For example, one probation officer reflected that treating substance use among youth is very important, “because if we don’t treat it now, they’re just going to keep using and that just causes more problems for them and their families.”

Community mental health respondents felt prepared to implement the EBP, and expressed interest in “seeing how any model that’s created… [seeing] if it actually works. If we’re following the model, is there success from it? That’s important.” Concordant with reflections from the qualitative interviews, results from the implementation leadership scale indicated that community mental health staff members felt that their supervisors were supportive (mean = 4.0, SD = 0.9; See Table 3), proactive (mean = 3.5, SD = 1.1), and knowledgeable (mean = 3.0, SD = 0.9) when it came to leading implementation of EBPs. Juvenile justice staff members also felt that their supervisors were broadly supportive (mean = 4.2, SD = 0.9), proactive (mean = 3.9, SD = 1.1), and knowledgeable (mean = 4.1, SD = 1.1) when it came to leading implementation of EBPs.

In terms of work behavior, respondents from both organizations expressed concerns about areas that were specific to their own realms of implementation. Community mental health clinicians reported skepticism about their ability to treat substance use and reported concerns that staff might not feel prepared or be sufficiently trained in providing treatment for substance use disorders. One clinic administrator reflected that “from a case manager perspective, we have staff, but not staff trained in [providing services for clients dealing with] substance abuse.” For juvenile justice implementation, respondents were skeptical of the effectiveness of screening procedures. While staff were positive about their own ability to implement screening procedures, many expressed significant skepticism about how “honest” youth would be when reporting substance use during intake procedures. Probation officers described their own strategies for attempting to facilitate honesty among youth (e.g., asking questions in open-ended ways, developing trust), but ultimately said “we’re going into this knowing kids are not always going to be honest, but we’re hopeful that through the process that we designed, they will be more comfortable.”

### Barriers and facilitators to collaboration

Given the above system-level differences between community mental health center and juvenile justice organizations, a number of issues were raised regarding effective collaboration. Differences in funding and structure appeared to manifest in communication difficulties and capacity concerns among staff. In particular, several juvenile respondents expressed concern that their community mental health counterparts would not have the service capacity to take on referrals, as had sometimes been the case in the past. A juvenile justice administrator reflected that “[probation officers] make a referral and then they tell us why they can’t serve that kid .... [having a] waitlist and not having enough providers are their typical reasons.” Respondents also expressed that community mental health clinicians were sometimes difficult to communicate with, with one probation officer reflecting that “I understand that a lot of [the community mental health clinicians] have big caseloads as well, so to write up a report like that each month is a lot. I’ve noticed they have gotten a little bit better.”

Staff at community mental health centers expressed concern regarding consistent referral sources and communication. A community mental health administrator expressed that when it came to getting referrals from juvenile justice, “we have to go and remind them, and we’ll get referrals, and then six months later, I have to go and remind them and we’ll get referrals, and so they are very good at the front part, but sustaining that pathway is, they just get distracted.” Regarding communication, one therapist reflected that “typically when we get referrals from probation they are blank, so there’s not a whole lot of information in general, not just with substance abuse, but family history, why the family’s involved, or why the adolescent is involved in probation and those sorts of things. So, we have to go by what the family’s telling us which may or may not be completely accurate.”

Staff from both organizations were also asked about factors that facilitated collaboration between juvenile justice and community mental health. The primary facilitator of collaboration and communication across the two organizations appeared to be shared attitudes about the importance of serving youth who struggled with substance use and the need for additional options for substance use treatment services. For example, one probation officer reflected that “much of the frustrations and skill deficits with kids lead to substance use, and lead them back to negative peers,” and that facilitating youth access to substance use treatment often leads to “changes in their behavior.” In concert with this, a case manager reported that “[his juvenile clients] start using in high school, junior high, and then as they get older, that becomes more of a problem with ... A bunch of them I know that we’ll be dealing with are already on probation, already having legal issues. I think that’s a really good thing to start out early.”

## Discussion

The primary purpose of this study was to identify barriers and facilitators to 1) implementation of evidence-based substance use practices in juvenile justice and community mental health organizations and 2) collaboration across organizations, both by examining these processes through the lens of the adopter-based innovation model. Findings highlighted how system-level structural and cultural differences across organizations pose both unique barriers and facilitators to implementation of EBPs. Differences between juvenile justice and community mental health also hinder effective collaboration between organizations, although similar shared perceptions and motivation regarding substance use facilitate collaboration.

### Implementation of evidence-based substance use practices

Regarding juvenile justice organizations, barriers and facilitators to implementation were identified through the lens of diffusion of innovations. With respect to organizational structure, juvenile justice staff have a high level of autonomy and independence in decision-making regarding cases. On the one hand, this emphasis on autonomy creates a constructive culture, allowing for ease of implementation of evidence-based screening as juvenile justice staff are less burdened with pre-existing mandatory requirements and have the flexibility to comfortably adapt to newly implemented substance use screening practices. On the other hand, the lack of centralized power or accountability through structured meetings could hinder sustainability or fidelity to the implemented screening procedures; for example, community mental health center staff noted that juvenile justice staff refer youth to their services when reminded, but have difficulty sustaining referrals. Nonetheless, juvenile justice staff voiced a commitment to EBPs and emphasized the importance of addressing substance use among youth.

In contrast to juvenile justice organizations, community mental health centers have more “red tape” (i.e., strict administrative procedures), less autonomy, and more centralized power. Case managers and workers described little autonomy in decision-making regarding cases and must adhere to requirements from state and federal agencies. They also have more administrative and clinical requirements that are monitored and necessary for funding. These existing requirements make it challenging to implement substance use treatment as staff have less flexibility in their work schedule and thus limited time to get adequately trained in evidence-based substance use treatment. Although this was not directly discussed in our interviews, community mental health centers also often have high levels of staff turnover that can negatively impact the implementation and sustainment of EBPs, as a steady flow of new staff members requires a large investment in training [40, 41]; in our study, community mental health center staff did express skepticism about having enough staff members trained in working with substance use disorders. The combination of these challenging factors has the potential to create a defensive culture in which community mental health staff feel unable to innovate and adopt new practices. Still, the more centralized power and accountability of staff to supervisors could serve as a potential facilitator to implementing evidence-based substance use treatment and also enhance fidelity to evidence-based treatment across the organization. Finally, respondents from community mental health centers all voiced strong commitment to EBPs and were in the process of receiving training in each intervention, but given the burden of unexpected challenges that implementation often places on providers, this commitment may change as implementation progresses.

### Collaboration

In addition to committing to EBP implementation, juvenile justice and community mental health centers must effectively collaborate in order to implement programs across organizations. Previous work [22] has identified key barriers and facilitators of collaboration between the two organizations. Kapp and colleagues [22] note that the lack of both formal service protocols and existing informal relationships present a barrier to positive communication between juvenile justice and community mental health organization. The two also have differing philosophies about the best way to approach justice-involved youth who struggle with substance use (e.g., attitudes about mandated treatment). Finally, existing strain on many community mental health centers (e.g., high caseloads, high clinician turnover rates) only serves to further strain the relationship between juvenile justice and community mental health. Our results are consistent with the previous finding that existing training with community mental health services makes collaboration difficult, but we did not find evidence of differing philosophies about the best way to approach justice-involved youth who struggle with substance use. In fact, our results suggest that shared views on the importance of addressing substance use may facilitate collaboration.

### Limitations

These findings show the importance of understanding organizational social context when implementing EBPs, however, this study does have some limitations. Although the themes described in this study reflect broad characteristics of juvenile justice or community mental health organizations that have been reported on in other studies [22], these findings may not generalize to all juvenile justice or community mental health organizations. In particular, all of the organizations in this study were located in rural or suburban areas; organizations in urban areas may experience different challenges when working together or implementing EBPs. Finally, the screening intervention that juvenile justice organizations planned to implement is less onerous than either of the treatment interventions, and there are considerable differences in the burden placed on community mental health organizations by the two treatment interventions (Teen Intervene and ENCOMPASS). Although both community mental health organizations planned to implement both interventions, responses may have been affected by the practice that staff were planning to implement.

### Implications

Our results indicate that while both community mental health and juvenile justice organizations express commitment to implementing EBPs, there may be a need for systems-level change to allow community mental health providers to increase capacity and bandwidth for providing evidence-based services. Still, evidence has shown that not only organizational-level but also individual-level factors influence the use of EBPs among community mental health clinicians [42]. Thus, smaller scale efforts may also show promise. One way to facilitate better collaboration across organizations may be to foster frank conversations between local juvenile justice and community mental health centers about service capacity, service needs, and how their organizations operate. Organizations may be able to make local changes to procedures that will enable them to work well together. Possible interventions to foster collaboration could include a learning collaborative model [43] that can foster the dissemination of EBPs across settings [44] and foster cross-organization collaboration [43]. In sum, collaboration across organizations with diverse goals, views and needs can be difficult. Seeking novel ways to foster collaboration and capitalizing on shared values (i.e., the importance of addressing youth substance use) among organizations is vitally important to improve outcomes among vulnerable youth [43].

## Conclusions

Participants from both juvenile justice and community mental health organizations endorsed a need to provide substance use disorder treatment for justice involved youth, and were committed to EBPs. However, community mental health respondents indicated skepticism about their own resources to effectively implement these services. Our results suggest that juvenile justice and community mental health organizations should find ways to increase collaboration in order to leverage resources for evidence-based substance use disorder treatment and improve implementation success.

## Supplementary information

**Additional file 1.** Interview Guides.

## Data Availability

The dataset analyzed during the current study is available from the corresponding author on reasonable request.

## Abbreviations

ILS: Implementation leadership scale
ORIC: Organizational readiness for change scale
ICS: Implementation climate scale
COREQ: Consolidated criteria for reporting qualitative research
EBP: Evidence-based practices
