# Supplementary material for: Organizational structure, climate, and collaboration between juvenile justice and community mental health centers: implications for evidence-based practice implementation for adolescent substance use disorder treatment
Source: BMC Health Serv Res. 2020 Oct 8;20:929. doi: 10.1186/s12913-020-05777-3 (PMC7545946; doi:10.1186/s12913-020-05777-3)
Supplement: Supplementary file 1 — Additional file 1. Interview Guides. [file 12913_2020_5777_MOESM1_ESM.docx]

**Appendix A. Interview Guides**

**Interview Guide for Juvenile Justice System Personnel: Pre-Implementation**

Part 1: Personal info:

*First, I would like to start with some background questions to learn more about you.*

1. Would you mind telling me a little bit more about your role at [organization]?
2. What is your favorite part of your job? *Tell me more.*
3. What is the most challenging part of your job? *Tell me more.*
4. *For Probation Officers only* - How are you currently supervised as a probation officer?
5. Have you ever tried group supervision? Do you have any thoughts about probation officer group supervision?

Part 2: Substance Use Treatment

*We are now going to shift the focus to questions about the substance use treatment options for justice involved youth, as well as your views related to substance use treatment.*

1. When [your organization] identifies youth as having/being at risk for substance abuse problems, what types of services do you refer them to*? Do you refer youth at all levels of risk (low, moderate, high) for substance use?*
2. What is your perspective on how effective your current referral options for teen substance use treatment options are? *Can you tell me more about why you feel this way?*
3. What are some potential treatment gaps that exist within your referral options for youth with substance use issues?
4. How important do you feel it is to address adolescent substance use?
   1. (If they think it is very important) What are you or your organization doing to address this issue?

Part 3: FB-JIP Implementation

*As mentioned earlier, our research team is working with county juvenile justice system to implement and test the screening, referral, and appropriate treatment program for youth with problematic substance use. Juvenile Justice personnel will screen youth for substance use and will aid the research team in referring youth who meet study criteria and consent to participate in the research study to the community mental health center for treatment.*

Can you describe your existing process for referring youth to mental health services?

How could the above referral process be improved?

What are some administrative challenges you foresee with screening youth for substance use? *For instance:*

**[Screening]** Do you feel comfortable using the CRAAFT measure to screen for substance use? *What are some of your thoughts about this process?*

*For Probation Officers only* - Let’s talk briefly about engagement with youth and families. Can you describe what parent engagement means to you for a youth on probation?

1. How do you engage parents in the probation process?
2. Is parent engagement important?

Part 4: Communication across systems and wrap up

*Thank you so much for your feedback so far. We just have one more section left before we wrap up. In this last section we will discuss your successes and challenges in working across systems.*

*Think about your experiences working with the community mental health system (identify partner organization by name, either Centerstone or Wabash Valley). This could include working with youth who have been referred to the community mental health center and communication with community mental health staff.*

1. Within your role at this organization, do you interact with the community mental health system? *If yes, how often? Probe for the type of interaction.*
2. What do you enjoy about [your interactions with the community mental health center?]
3. What are some challenges you have experienced in working with community mental health center staff?
4. Do you have any suggestions on how to improve the interaction [and/or communication] between your organization and the community mental health system?

*Now, think about your past experiences working with researchers and/or academic institutions.*

1. Can you describe the type of experiences you have had with the research field? What have been some positive experiences? How about some negative experiences?
2. In your experience, what are some unique challenges that CMHCs face when working with researchers? (To probe differently… What are some things that researchers tend to forget to consider when working with JJ Centers?)

*We are almost finished! Just two more questions to wrap up. When you think about this research study:*

1. What are some aspects of the project that you are concerned about?
2. What are some aspects of the project that you are most excited about?

**Interview Guide for CMHC System Personnel: Pre-Implementation**

# Part 1: Personal info

*First, I would like to start with some background questions to learn more about you.*

1. Tell me a little bit more about your role at [community mental health center]?
2. What is your favorite part of your job? *Tell me more.*
3. What is the most challenging part of your job? *Tell me more*

Thank you for sharing.

# Part 2: Substance Use Treatment

*We are now going to shift the focus to questions about the substance use treatment options your organization currently provides, as well as your views related to substance use treatment.*

1. What types of services does your organization currently provide to adolescents with substance use problems? *Do you provide any services for youth at the low to moderate risk category?*
2. What is your perspective on how effective your current teen substance use treatment options are? *Can you tell me more about why you feel this way?*
3. What are some potential treatment gaps that exist within your organization for adolescents with substance use issues?
4. How important do you feel it is to address adolescent substance use?
   - (If they think it is very important) What are you or your organization doing to address this issue?

## Part 3a: Referral Process

*As mentioned earlier, our research team is working with [Wayne or Tippecanoe] county juvenile justice system to implement and test the screening, referral, and appropriate treatment program for youth with problematic substance use. Youth who meet study criteria and consent to participate in the research study, will be referred by the research team to your agency for treatment.*

1. Can you describe your existing referral process for youth involved in juvenile justice?
2. How could the above referral process be improved?

## Part 3b: ENCOMPASS Intervention

*In this study, youth who are assessed at the highest risk for substance use will be directed to ENCOMPASS, a comprehensive, outpatient substance use treatment program designed for adolescents.*

1. What type of feedback have you heard from clinicians and administrators within your organization about ENCOMPASS? *Are they excited about the program?*
2. Are there any systems/advantages within your organization that might facilitate the ENCOMPASS program?
3. What are some administrative challenges you foresee with the ENCOMPASS program? *For instance:*

- [**Drug Screening]:** ENCOMPASS participants have to complete a urine drug screen prior to their session with a therapist to track their progress. *Is this something your organization already does? If not, how feasible would it be to implement this change?*
- **[Contingency Management]:** ENCOMPASS includes a contingency management prize program to promote abstinence, treatment compliance and completion of pro-social non-drug related community activities. *What are some of your thoughts about the prize program?*

## Part 3c: Brief Interventions:

*Youth who are assessed at low or moderate risk for substance abuse will be randomized to one of two brief interventions, Teen Intervene or Family Checkup. Both of these interventions can be completed in 3-6 one-hour sessions facilitated by a trained bachelor’s level case manager. The major difference between the interventions, is that one is focused primarily on the teen, while the second intervention is focused on the family. These interventions both use motivational interviewing techniques and have been shown to reduce substance use among youth. We are interested in learning if these interventions have positive outcomes for youth involved in the justice system.*

1. What are some administrative challenges that you foresee with the brief interventions? (Only ask administrators and managers)
   1. *Medicaid reimbursement, billing for DCS (right now we can’t bill DCS for this service?*
   2. *How has the organization thought through changes to the case manager’s schedule to allow them to complete the intervention?*
2. Are there any systems/advantages within your organization that might facilitate the ENCOMPASS program?

*One of the unique features of the brief interventions is they can be led* by *a trained bachelor’s level case manager. (If interviewing a case manager, ask about their own feelings/opinions)*

1. How feasible do you think it will be for bachelors-level case managers to provide a brief intervention to youth in the low to moderate risk category for substance use problems? *What are some specific challenges you might foresee?*
   1. How will implementing these interventions change case managers’ schedules? Do you anticipate any challenges related to the time case managers will be spending on these interventions?
2. Are there any elements of these interventions that will be completely new to case managers? Is there anything that the research team can do to mitigate the challenges we’ve discussed?

# Part 4: Communication across systems and wrap up

*Thank you so much for your feedback so far. We just have one more section left before we wrap up. In this last section we will discuss your successes and challenges in working across systems.*

*Think about your experiences working with the juvenile justice system. This could include working with clients who have been referred through the justice system and communication with probation officers.*

1. Within your role at this organization, do you interact with the justice system? *If yes, how often? Probe for the type of interaction.*
2. What do you enjoy about [your interactions with the justice system?]
3. What are some challenges you have experienced in working with probation officers or other justice personnel?
4. Do you have any suggestions on how to improve the interaction [and/or communication] between your organization and the juvenile justice system?

*Now, think about your past experiences working with researchers and/or academic institutions.*

1. Can you describe the type of experiences you have had with the research field? What have been some positive experiences? How about some negative experiences?
2. In your experience, what are some unique challenges that CMHCs face when working with researchers? (To probe differently… What are some things that researchers tend to forget to consider when working with CMHCs?)

*We are almost finished! Just two more questions to wrap up. When you think about this research study:*

1. What are some aspects of the project that you are concerned about?
2. What are some aspects of the project that you are most excited about?
